# A Chinese Herbal Preparation, Xiao-Er-An-Shen Decoction, Exerts Neuron Protection by Modulation of Differentiation and Antioxidant Activity in Cultured PC12 Cells

**DOI:** 10.1155/2018/8670421

**Published:** 2018-05-03

**Authors:** Zhonggui Li, Kelly Y. C. Lam, Xiaoyan Liu, Airong Qi, Ping Yao, Tina T. X. Dong, Tiegang Yi, Karl W. K. Tsim, Jianping Chen

**Affiliations:** ^1^Shenzhen Key Laboratory of Hospital Chinese Medicine Preparation, Shenzhen Traditional Chinese Medicine Hospital, Guangzhou University of Chinese Medicine, Shenzhen, China; ^2^Division of Life Science and Center for Chinese Medicine, The Hong Kong University of Science and Technology, Kowloon, Hong Kong

## Abstract

Xiao-Er-An-Shen Decoction (XEASD), a Chinese herbal formula, has been used in clinic for treating insomnia and mental excitement in children and adolescents. However, less of scientific data supports its effectiveness in clinic. Here, we aim to study the role of XEASD in regulating neuron differentiation and antioxidant activity. An HPLC-MS was used to chemically standardize herbal extract of XEASD. The standardized herbal extracts of XEASD (0.3–3.0 mg/mL) were applied onto cultured PC12 cells for 48 hours. The treatment with XEASD extract induced neurite outgrowth of PC12 cells in a dose-dependent manner, having the highest response by ~50% of differentiated cells. Application of XEASD extract dose dependently stimulated expressions of NF68, NF160, and NF200 in cultured PC12 cells. Furthermore, XEASD activated the phosphorylation of cAMP responsive element binding protein on PC12 cells, the effect of which was blocked by H89, a protein kinase A inhibitor. Moreover, XEASD showed free radical scavenging activity and stimulated the transcriptional activity of ARE. These results supported the neurobeneficial effects of XEASD in the induction of neurite outgrowth and protection against oxidative stress and could be useful for neurological diseases, in which neurotrophin deficiency and oxidation insult are involved.

## 1. Introduction

Sleep disturbances are widely spread in children and adolescents, and the prevalence of sleep-related problems was estimated to be ~43% [1]. Insomnia is characterized by a difficulty in falling asleep and/or staying asleep, which is the most common sleep disorder with high rate of serious conditions [2]. It was reported that people with insomnia have a twofold risk to develop depression compared to people with no difficulty in falling asleep [3]. Moreover, it has been estimated that 90% of patients with depression complain about their sleep quality [4]. Sleep disorders are linked with potential suicidal behaviors in depressed youth [5]. Thus, sleep problems like insomnia should be taken as major concern among parents and doctors. Hypnotics are primarily used in clinic to treat insomnia, yet the treatment of this is accompanied by many side effects. This is not an ideal therapeutic regimen for children and adolescents. Therefore, there is a need to develop an alternative medicine for the treatment of insomnia in children and adolescents.

Neurogenesis is the process by which neurons are generated, including cell proliferation, survival, maturation, and differentiation [6]. The findings suggested that disturbance of sleep can lead to a reduction of neurogenesis. And a decrease in neurogenesis was also considered as one of the critical factors contributing to the pathophysiology of depression [7]. Brain development in children and adolescence is foundation upon which the brain continues developing, the impairment of which can result in a fragile and unreliable foundation. Taking together, we propose that enhancement of neurogenesis could be one of the effective approaches in improving sleep problems and further preventing the development of depression for youth.

Xiao-Er-An-Shen Decoction (XEASD), a Chinese herbal formula, has been clinically prescribed to treat insomnia and mental excitement in children and adolescents, which possesses the efficacies of nourishing the brain to tranquilize and dispelling phlegm to settle fright. However, less is known about the molecular mechanism of XEASD in supporting its brain benefit. In our current studies, we investigated whether XEASD possessed neurogenesis role in neuronal cells. The effect of XEASD on neuron differentiation, an important stage of neurogenesis, was firstly evaluated. We determined the neurite outgrowth and neurofilament expression in neuronal PC12 cells, a wide model for the study of neuronal differentiation. In addition, the antioxidative role of XEASD was revealed to prevent the impairment of neurogenesis under oxidative stress.

## 2. Materials and Methods

### 2.1. Chemical and Drugs

XEASD was kindly provided by Pharmaceutical Department of Shenzhen Traditional Chinese Medicine Hospital. XEASD mainly consists of night herbs and the mixed proportion of respective herb is illustrated in Table 1. Liquiritin (1), calycosin 7-O-*β*-glucoside (2), ammonium glycyrrhizinate (3), naringin (4), 3,6′-disinapoylsucrose (5), hesperidin (6), neohesperidin (7), and astragaloside IV (8) were purchased from National Institutes for Food and Drug Control (Beijing, China). The purities of all these chemicals were over 98%. HPLC grade acetonitrile was purchased from Merck (Darmstadt, Germany), and ultrapure water was prepared using a Milli-Q purification system (Molsheim, France). Other reagents were of analytical grade.

### 2.2. Chromatographic Conditions and Instrumentation

A validation HPLC method was applied onto a Shimadzu (Kyoto, Japan) LC-20AT system. The herbal extract was separated on Agilent ZORBAX SB-C18 (250 mm × 4.6 mm, 5 *μ*m) column. The mobile phase was composed of acetonitrile (A) and 10 mmol/L ammonium acetate (B) using the following gradient program: 0–0.01 min, 15% A; 0.01–5 min, 15%–19% A; 5–22 min, 19%–35% A; 22–35 min, 35%–90% A; the flow rate was 0.8 ml/min; the injection volume was 5 *μ*l. A Shimadzu mass spectrum (LC-2020) equipped with an ESI ion source was performed in both positive and negative modes, and the selected ion monitoring was employed. The drying gas temperature was 350°C; drying gas flow was 1.5 L/min; nebulizer pressure was 35 psi; capillary voltage was 3500 V. Shimadzu Mass Workstation software was used for data acquisition and processing.

### 2.3. Cell Culture

Pheochromocytoma PC12 cells, a cell line derived from rat adrenal medulla, were purchased from American Type Culture Collection (ATCC, Manassas, VA). The cells were cultured in Dulbecco's modified Eagles medium (DMEM) supplemented with 6% fetal bovine serum (FBS), 6% horse serum (HS), 100 U/mL penicillin, and 100 *μ*g/mL streptomycin at 37°C in a water-saturated 7.5% CO_2_ incubator. Cultured medium was changed with fresh one every other day. Reagents for cell cultures were obtained from Invitrogen Technologies (Carlsbad, CA).

### 2.4. Neurite Outgrowth Assay

The assessment of neurite outgrowth in PC12 cells was performed according to previous report [8]. In brief, Cultured PC12 cells were treated with drugs for 48 hours and changed with medium containing XEASD every 24 hours. Nerve growth factor (NGF; Alomone Labs; Jerusalem, Israel) at concentration of 50 ng/mL was employed as a positive control. Cells were fixed with ice-cold 4% paraformaldehyde immediately after collection. A light microscope (Zeiss Group, Jena, Germany) equipped with a phase-contrast condenser, 10x objective lens, a digital camera, and SPOT imaging software was employed to capture and analyze the neurite presence and neurite length, and approximately 100 cells were counted from more than 10 randomly chosen visual fields per each culture. The cells were defined as differentiated if one or more neurites were longer than the diameter of cell body and also classified according to their neurite length in <15 *μ*m, 15–30 *μ*m, and >30 *μ*m.

### 2.5. Sodium Dodecyl Sulfate Polyacrylamide Gel Electrophoresis (SDS-PAGE)

To investigate neurofilament expression, PC12 cells (8 × 10^4^ cells/mL) were seeded onto 12-well plates in normal serum medium for 24 hours, and then medium was transferred to low serum (1% FBS, 1% HS, 100 U/mL penicillin, and 100 *μ*g/mL streptomycin) as indicated for 3 hours prior to exposure XEASD. After 48 hours of treatment, the cultures were collected in high salt lysis buffer (1 M NaCl, 10 mM HEPES, pH 7.5, 1 mM EDTA, 1 mM EGTA, and 0.5% Triton X-100), followed by centrifugation at 16,100 ×g for 10 mins at 4°C. Total protein content was determined by Bradford's method with a kit from Bio-Rad Laboratories (Hercules, CA). Samples of equal amount of total protein were added with 2x direct lysis buffer (0.125 M HCl, pH 6.8, 4% SDS, 20% glycerol, 2% 2-mercaptoethanol, and 0.02% bromophenol blue) and boiled for 10 mins before being separated on 8% gel electrophoresis.

### 2.6. Protein Phosphorylation on PC12 Cells

PC12 cells were seeded onto 12-well plate. After the degree of confluence was over 90 percentage, the culture medium was changed to DMEM medium without serum over 5 hours. The cells were treated with XEASD or NGF at different time points in the absence or presence of H89 (0 min, 5 min, 10 min, and 30 min). Then, the cells were harvested, digested with 200 *μ*L of 2x direct lysis buffer, and boiled for 10 min before undergoing 8% gel electrophoresis.

### 2.7. Western Blot Analysis

Following the electrophoresis, proteins running on an 8% SDS-PAGEs were transferred to the nitrocellulose membrane, using a Mini Trans-Blot® cell at 40 V and 0.1 A for 16 hours in 1x transfer buffer with 24 mM Tris, 192 mM glycine, 15% ethanol, and 0.1% SDS. Transfer and equal loading of the samples were confirmed using Ponceau-S staining. After being blocked with 5% fat-free milk in Tris-buffer saline/0.1% Tween 20 (TBS-T), the membranes were incubated in the primary antibody diluted in 2.5% fat-free milk in TBS-T at 4°C overnight. The primary antibodies used were antineurofilament 200 (NF200; 1 : 1,000, Sigma), anti-NF160 (1 : 2,500, Sigma), anti-NF68 (1 : 2,500, Sigma), and anti- GAPDH (1 : 10,000; Abcam Ltd., Cambridge, UK). Then, the membrane was rinsed with TBS-T and incubated for 2 hours at the room temperature in horseradish peroxidase- (HRP-) conjugated anti-mouse secondary antibody (Invitrogen) diluted in the 2.5% fat-free milk in TBS-T. The immune complexes were visualized using the enhanced chemiluminescence (ECL) method (GE Healthcare, Piscateway, NJ). The densitometric analysis of the bands in various samples was performed on an image analyzer.

### 2.8. DNA Construction and Transfection

The vector, pGL4.37 [*luc2P*/ARE/Hygro], contains four copies of an antioxidant response element (ARE, 5′-TAGCTTGGAA ATGACATTGC TAATGGTGAC AAAGCAACTT T-3′) having a downstream luciferase reporter gene* luc2P (Photinus pyralis)* (Promega Corporation, Madison, WI). Cultured PC12 cells were transiently transfected with pARE-Luc using Lipofectamine 3000 (Thermo Fisher Scientific Inc, Waltham, MA), according to the manufacturer's guidelines. The cells were ready for experiments after 24 hours of transfection.

### 2.9. Luciferase and Protein Assays

After drug treatment, luciferase assay was analyzed by a commercial kit (Tropix Inc., Bedford, MA). Briefly, cultures were digested by a buffer containing 100 mM potassium phosphate buffer (pH = 7.8), 0.2% Triton X-100, and 1 mM dithiothreitol. The luminescent reaction was measured in a Glomax™ 96 Microplate Luminometer, and the activity was expressed as absorbance (up to 560 nm) per mg of protein. Protein concentrations were determined routinely by Bradford's method with a kit from Bio-Rad Laboratories (Hercules, CA).

### 2.10. DPPH Radical Scavenging Assay

DPPH solution (100 *μ*g/ml, 2,2-diphenyl-1-picrylhydrazyl, Sigma) was prepared with methanol (HPLC grade). Fifty *μ*L of extracts with different concentration together with 150 *μ*L of DPPH solution was added to each well of 96-well microplate. After incubating for 30 mins at room temperature avoiding light, the absorbance was recorded at 495 nm for each sample tested against methanol blank using a spectrophotometer. The DPPH radical scavenging effect was expressed as % scavenging relative to control (without samples).

### 2.11. Statistical Analysis

Statistical tests were analyzed using *T*-Test (version 13.0, SPSS, IBM Corporation, Armonk, NY). Statistically significant changes were classified as significant (*∗*) where* p* < 0.05, more significant (*∗∗*) where* p* < 0.01, and highly significant (*∗∗∗*) where* p* < 0.001.

## 3. Results

### 3.1. Chemical Standardization of XEASD

**Figure 1:**
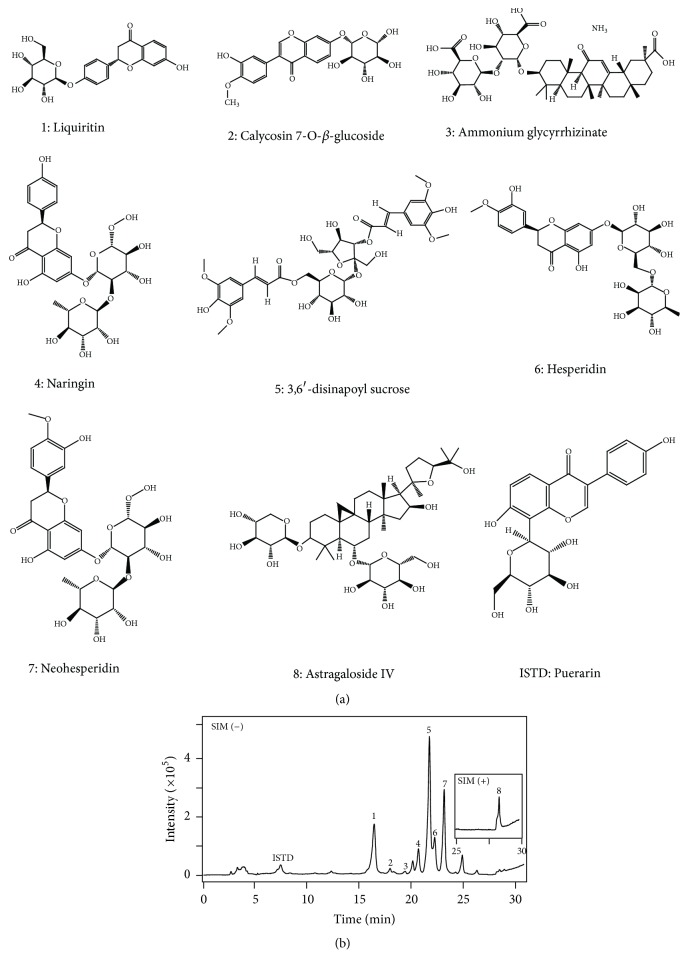
*The representative HPLC-MS chromatogram of XEASD*. (a) Structures of chemical markers identified in XEASD extract, including liquiritin (1), calycosin 7-O-*β*-glucoside (2), ammonium glycyrrhizinate (3), naringin (4), 3,6′-disinapoylsucrose (5), hesperidin (6), neohesperidin (7), astragaloside IV (8), and puerarin (internal standard, ISTD). (b) The representative LC-MS chromatogram of XEASD extract. The LC condition was indicated in the Materials and Methods. The denotations from 1 to 8 in the chromatogram correspond to the chemical markers as shown in (a). The identification of the chemical markers was analyzed by a MS detector in a negative mode except astragaloside IV (8) in a positive mode. Representative chromatograms were shown, *n* = 3.

An HPLC-MS method was developed to reveal the chemical profile and quantify the main ingredients of XEASD. Eight chemical markers were identified in XEASD extract (Figure 1(a)). A typical LC-MS profile was developed for XEASD extract (Figure 1(b)), which was employed as a parameter for the identification of XEASD. Besides, eight chemical components were quantified in the XEASD extract defining the minimum requirement for 1 g of dried powder of standardized XEASD, that is, liquiritin (0.13 mg/g), calycosin 7-O-*β*-glucoside (0.23 mg/g), ammonium glycyrrhizinate (0.26 mg/g), naringin (1.80 mg/g), 3,6′-disinapoylsucrose (0.11 mg/g), hesperidin (0.12 mg/g), neohesperidin (0.65 mg/g), and astragaloside IV (0.05 mg/g). The extract being used in this study reached the aforementioned requirements, which ensured the repeatability of biological results.

### 3.2. XEASD Induces Neurite Outgrowth and Neurofilament Expression of PC12 Cells

**Figure 2:**
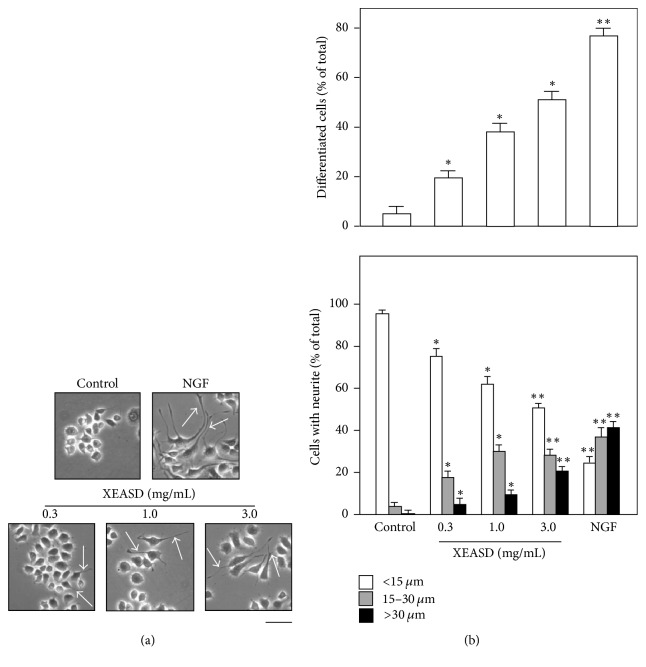
*XEASD induces neurite outgrowth of cultured PC12 cells*. (a) Cultured PC12 cells were treated with XEASD extract at various concentrations (0.3–3.0 mg/mL) for 48 hours. NGF (50 ng/mL) served as the positive control. After treatment, cells were fixed with ice-cold 4% paraformaldehyde, and then the neurite outgrowth was measured under microscope. Representative images were shown,* n* = 5. Bar = 20 *μ*m. Arrowheads indicate cell neurite. (b) To quantify the differentiation effect, the % of differentiated cell numbers (upper panel) and length of neurite (lower panel) were calculated as indicated in the Materials and Methods. The cells were defined as differentiated if one or more neurites were longer than the diameter of cell body and also classified according to their neurite length in <15 *μ*m, 15–30 *μ*m, and >30 *μ*m. Data are expressed as % of cells in 100 counted cells. Mean ± SEM;* n* = 4. Statistical comparison was made with the control; ^*∗*^*p* < 0.05; ^*∗∗*^*p* < 0.01.

**Figure 3:**
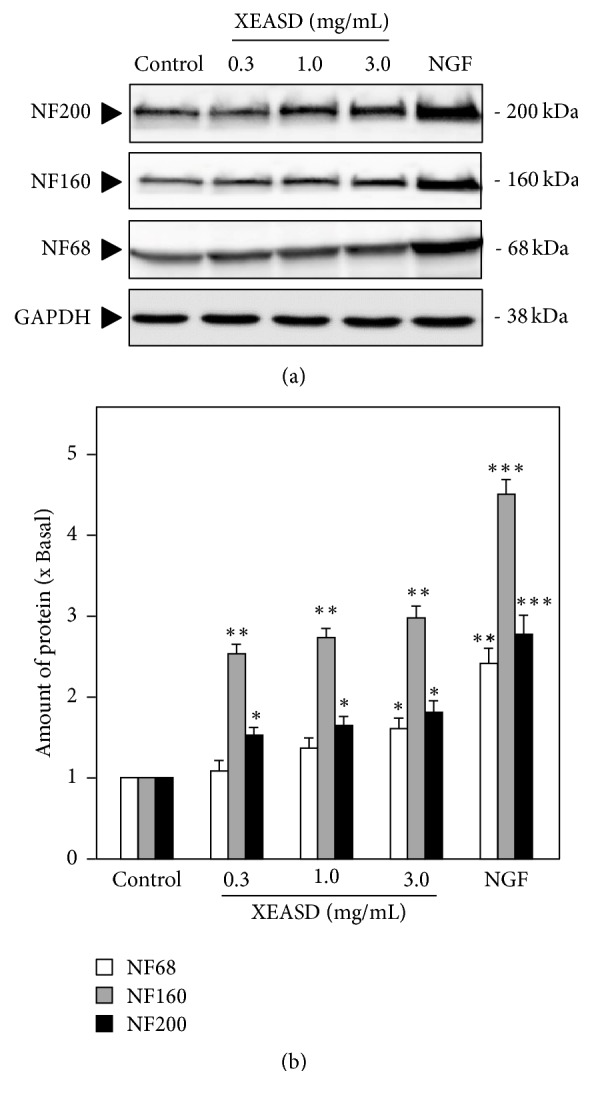
XEASD stimulates neurofilament expression of cultured PC12 cells. (a) XEASD extracts (0.3–3.0 mg/mL) were applied onto cultured PC12 cells for 48 hours. The cell lysates were collected to determine the expressions of NF68, NF160, and NF200. NGF (50 ng/mL) served as the positive control. GAPDH served as a loading control. (b) Quantification plot was shown in histograms. Values are expressed as the fold of increase to basal reading (untreated culture, set as 1). Mean ± SEM;* n* = 4. Statistical comparison was made with the control; ^*∗*^*p* < 0.05; ^*∗∗*^*p* < 0.01; ^*∗∗∗*^*p* < 0.001.

To study the trophic effect of XEASD on PC12 differentiation, cultured PC12 cells were treated with XEASD extract for 48 hours before examination under light microscope. As indicated in Figure 2(a), it was observed that neurites were produced from the cell bodies of PC12 cells by XEASD treatment. To quantify the morphological change, that is, differentiated cells and neurite length, the results showed that after the treatment with XEASD extract at various concentrations on PC12 cells for 48 hours, the differentiated cells at highest response were calculated to ~50% of the total cells in cultures (Figure 2(b), upper panel). As for neurite length, application of XEASD extract onto PC12 cells also increased the number of cells possessing neurite length at 15–30 *μ*m and >30 *μ*m (Figure 2(b), lower panel). Apart from the morphological observation, the expression of neurofilaments including NF68 (at ~68 kDa), NF160 (at ~160 kDa), and NF200 (at ~200 kDa) was determined by a biochemical analysis to evaluate cell differentiation. Application of XEASD extract at various concentrations (0.3, 1.0, and 3.0 mg/mL) stimulated the expressions of NF68, NF160, and NF200, with the highest induction, ~0.5-fold, 2-fold, and 3-fold, respectively (Figures 3(a) and 3(b)). NGF served as positive control, which could induce neurite outgrowth and the expression of neurofilament in PC12 cells.

**Figure 4:**
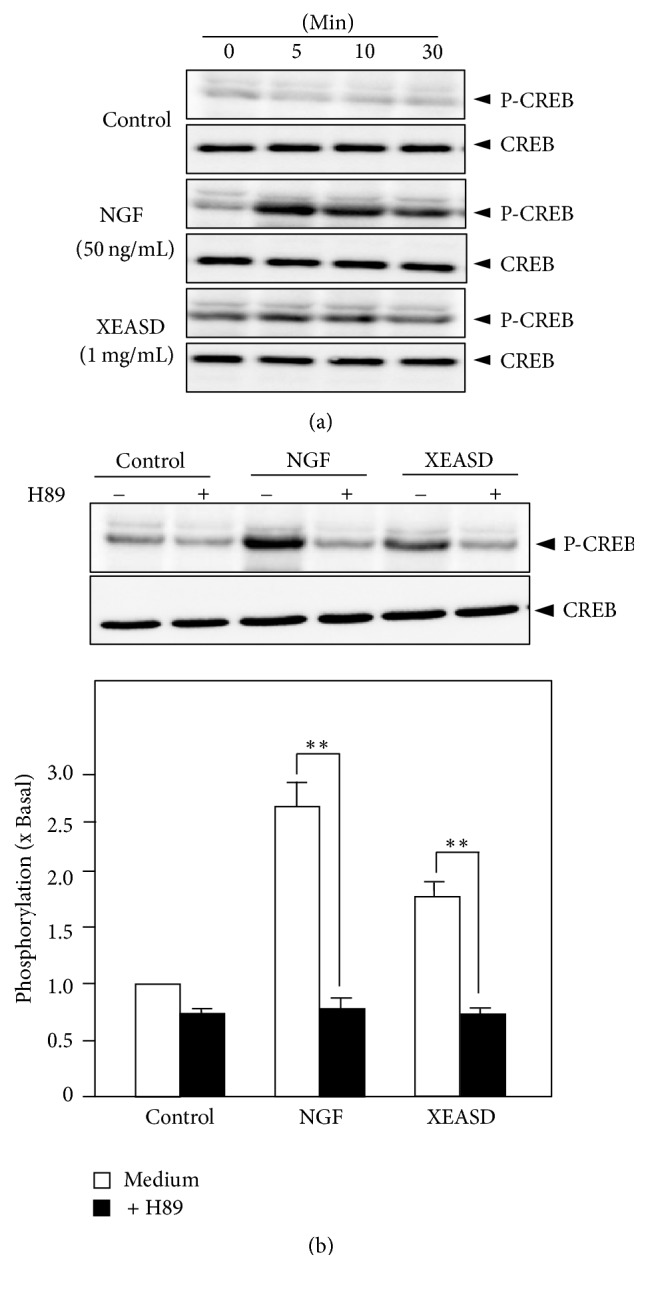
XEASD induces CREB phosphorylation on PC12 cells. (a) Cultured PC12 cells were serum starved over 5 hours before the treatment with XEASD extract (1 mg/mL) or NGF (50 ng/mL) for different time. Total CREB and phosphorylated CREB (both at ~ 43 kDa) were determined by using specific antibodies. (b) Cultured PC12 cells with serum starvation for over 5 hours were pretreated with or without H89 (5 *μ*M; a PKA inhibitor) for 3 hours prior to the treatment with NGF (50 ng/mL) and XEASD (1.0 mg/mL) for 10 minutes. Total CREB and phosphorylated CREB were determined by using specific antibodies (upper panel). Quantification plot was indicated in histograms (lower panel). Values are expressed as the fold of change (x Basal) against the control (no treatment; set as 1). Mean ± SEM;* n* = 5. Statistical comparison was made with the H89-treated group; ^*∗∗*^*p* < 0.01.

The nuclear transcription factor cAMP responsive element binding protein (CREB) plays a vital role in neuronal differentiation on PC12 cells [9]. The activation of CREB by phosphorylation recruiting the coactivator CREB-binding protein allows its binding onto the promoter and further induces target gene transcription that results in neuronal differentiation. Hence, the inductive role of XEASD in activating the phosphorylation of CREB was studied. As shown in Figure 4(a), XEASD extract stimulated the phosphorylation of CREB at ~43 kDa, and its activation could be fully blocked by H89 (Figure 4(b)).

### 3.3. Antioxidant Activity of XEASD

**Figure 5:**
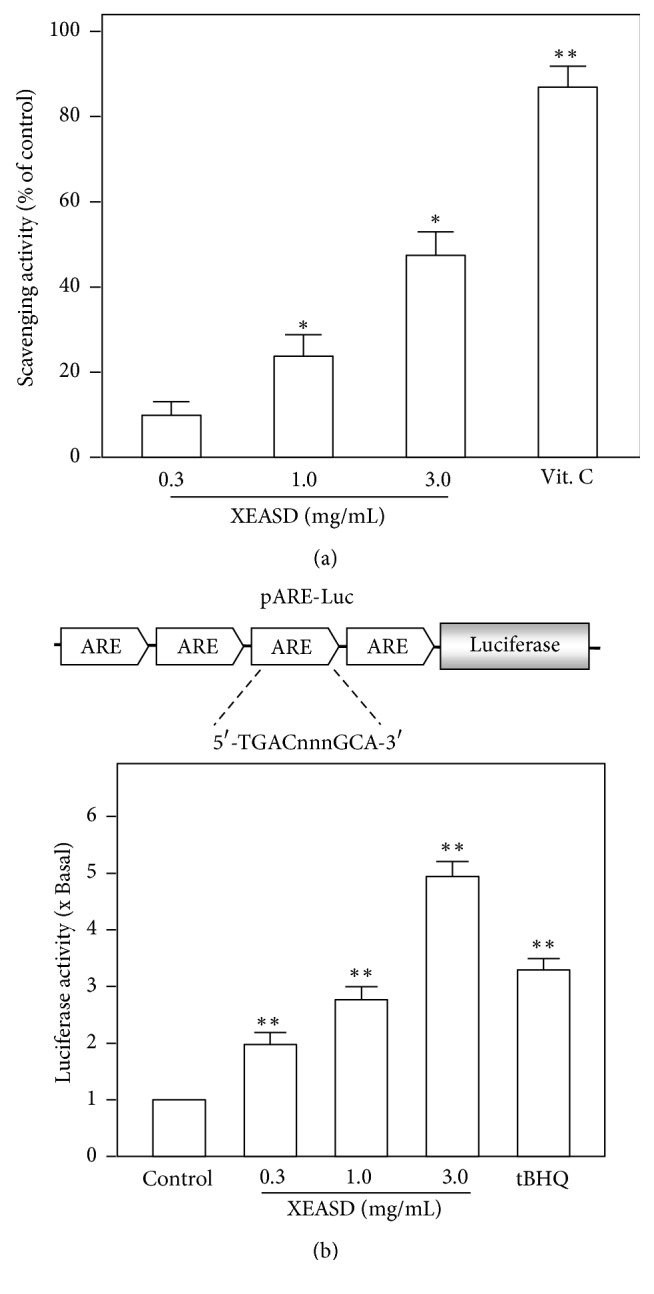
Antioxidation activities of XEASD. (a) An aliquot of 50 *μ*L of extracts and vitamin C (Vit. C) was added to a 150 *μ*L DPPH solution. After incubating for 30 min, the decrease in the absorbance of the mixture was determined at 495 nm. The DPPH radical scavenging effect was expressed as % scavenging relative to control (without samples). (b) A luciferase reporter containing four AREs and a downstream luciferase reporter gene, namely, pARE-Luc, was used as a study tool (upper panel). Cultured PC12 cells, transfected with pARE-Luc, were treated with XEASD extracts (0.3–3.0 mg/mL) for 24 hours. The cell lysates were subjected to luciferase assay. Values are expressed as the fold of increase to basal reading (untreated culture), and they are in mean ± SD, where *n* = 4, each with triplicate samples. Statistical comparison was made with the control; ^*∗*^*p* < 0.05; ^*∗∗*^*p* < 0.01.

The DPPH assay is widely used in analyzing the radical scavenging activity known to be involved with oxidation. Thus, the DPPH radical scavenging activity of XEASD was measured. The results indicated that XEASD extract exhibited scavenging free radical activities, and a dose-dependent manner was observed in reactions of DPPH radical with extracts (0.3–3.0 mg/mL) (Figure 5(a)). Vitamin C (Vit. C), a commercial antioxidant, served as a positive control. Furthermore, to study the transcriptional activity of ARE, PC12 cells were transfected with a luciferase reporter construct (pARE-Luc), containing four AREs derived from the promoter of antioxidant defense genes and having a downstream luciferase reporter gene (Figure 5(b), upper panel). The results demonstrated that application of XEASD extract stimulated the luciferase activity in a dose-dependent manner, with highest response at about 5-fold increase (Figure 5(b), lower panel). tBHQ treatment was used to authenticate the activation of pARE-Luc, which served as a positive control here (Figure 5).

## 4. Discussion

Neurogenesis is a process known to produce neurons in the brain, by which neuronal differentiation is a vital stage in developing neurons. During neuron differentiation, the axon and neurite of neuron cell are promoted so as to connect with other neurons to form synapse [10]. Morphological observation including determination of neurite length and calculation of differentiated cell number is widely used to evaluate neuronal differentiation of PC12 cells [11]. Besides, the expressions of neurofilaments including three kinds of molecular weight: 68 KD, 160 KD, and 200 KD were detected [12]. In our current study, application of XEASD could stimulate the neurite outgrowth of PC12 cells morphologically. In parallel, treatment with XEASD notably induced protein expression of NF160 and NF 200, but to a lesser extent in NF68. Generally, NF68 and NF160 are expressed for the early stage of the differentiation, while NF200 is for the mature stage. Hence, the role of XEASD in neuronal differentiation could be involved in the entire process at both early and late stages. Our further study also demonstrated that XEASD could induce the phosphorylation of CREB. We therefore proposed that PKA-CREB signaling was involved in XEASD-induced neuronal differentiation of PC12 cells.

Currently, induction of neuronal differentiation has been considered as a target for development of antidepressants [10]. Neurotrophin deficiency is reported to involve various neurodegenerative diseases. In depression patient, the neurotrophic factor secretion in the brain was found to reduce, resulting in malfunction of neuron survival, growth, and differentiation [13]. According our findings, XEASD may exert effect of NGF-like or neurotrophic factor stimulating agent in mediating neuronal differentiation of PC12 cells, supporting its application as a potential antidepressant.

On the other hand, oxidative stress is one of the major causes of neuronal cell death in many neurological disorders, that is, depression. Higher animals have developed elaborate defense mechanism, including phase II detoxification enzymes and antioxidant proteins to overcome these insults [14]. Antioxidant response element (ARE) signaling has been demonstrated to play an important role in protecting cells against oxidative stress [15]. We speculated that XEASD possessed free radical scavenging activity. Moreover, XEASD was able to stimulate the transcriptional activity of ARE in cultured PC12 cell. Due to the presence of ARE located on promoters of the defense genes [16], we proposed that the stimulation of the ARE signaling by XEASD may further induce the defense gene expressions of PC12 cells.

XEASD contained numerous herbs, and some of them as well as its major ingredients had been found to possess neurobeneficial effects. Acori Tatarinowii Rhizoma is widely employed clinically within a TCM formula for mental health. Volatile oils were considered as the major active ingredients of Acori Tatarinowii Rhizoma, which had been reported to induce neuronal differentiation [13]. 3,6′-Disinapoyl sucrose found from Polygalae Radix exerted antidepressant-like effect in depressive animal models [17]. Besides, euxanthone isolated from Polygalae Radix was also shown to possess a marked stimulatory action on neurite outgrowth [18]. Triterpenoids from Poria could regulate the expression of 5-HT3A receptors in Xenopus oocytes [19]. Flavanoids, such as naringin, hesperidin, and neohesperidin, from Aurantii Fructus and Citri Reticulatae Pericarpium were found to possess neuroprotective effects [20–22]. We hypothesized that the active ingredients in XEASD may exert the neuronal differentiation and antioxidant properties. To support this notation, some of the aforesaid components such as 3,6′-disinapoyl sucrose, naringin, hesperidin, and neohesperidin were identified and quantified within the extract of XEASD by LC-MS analysis, the effects of which need to be further studied.

## 5. Conclusions

XEASD could induce neurite outgrowth and stimulate the expression of neurofilament in PC12 cells, which supported the enhancement of neurogenesis by XEASD. Besides, XEASD protected cells against oxidative stress in regulating the transcriptional activity of ARE. In conclusion, XEASD could be useful for neurological diseases, in which neurotrophin deficiency and oxidation insult are involved, that is, insomnia and depression. The current studies also provided evidences supporting the clinical usage of XEASD for the treatment of insomnia in children and adolescents.

## Figures and Tables

**Table 1:** The composition and proportion of herb in XEASD.

Botanical name	Herbal name	Chinese name	Dosage
*Polygala tenuifolia* Willd.	Polygalae Radix	Yuan-Zhi	16 g
*Astragalus membranaceus* (Fisch.) Bge. var. *mongholicus* (Bge.) Hsiao	Astragali Radix	Huang-Qi	16 g
*Acorus tatarinowii* Schott	Acori Tatarinowii Rhizoma	Shi-Chang-Pu	25 g
*Citrus reticulate* Blanco	Citri Reticulatae Pericarpium	Chen-Pi	8 g
*Alpinia oxyphylla* Miq.	Alpiniae Oxyphyllae Fructus	Yi-Zhi	16 g
*Citrus aurantium* L.	Aurantii Fructus	Zhi-Qiao	16 g
*Pinellia ternata* (Thunb.) Breit.	Pinelliae Rhizoma	Ban-Xia	16 g
*Notopterygium incisum* Ting ex H. T. Chang	Notopterygii Rhizoma et Radix	Qiang-Huo	8 g
*Glycyrrhiza uralensis* Fisch.	Glycyrrhizae Radix et Rhizoma	Gan-Cao	6 g

## References

[1] Kim D. S., Lee C. L., Ahn Y. M. (2017). Sleep problems in children and adolescents at pediatric clinics.

[2] Meltzer L. J., Johnson C., Crosette J., Ramos M., Mindell J. A. (2010). Prevalence of diagnosed sleep disorders in pediatric primary care practices.

[3] Baglioni C., Battagliese G., Feige B. (2011). Insomnia as a predictor of depression: a meta-analytic evaluation of longitudinal epidemiological studies.

[4] Tsuno N., Besset A., Ritchie K. (2005). Sleep and depression.

[5] Lopes M., Boronat A. C., Wang Y., Fu-I L. (2016). Sleep complaints as risk factor for suicidal behavior in severely depressed children and adolescents.

[6] Meerlo P., Mistlberger R. E., Jacobs B. L., Heller H. C., McGinty D. (2009). New neurons in the adult brain: the role of sleep and consequences of sleep loss.

[7] Mueller A. D., Pollock M. S., Lieblich S. E., Epp J. R., Galea L. A. M., Mistlberger R. E. (2008). Sleep deprivation can inhibit adult hippocampal neurogenesis independent of adrenal stress hormones.

[8] Xu S. L., Choi R. C. Y., Zhu K. Y. (2012). Isorhamnetin, a flavonol aglycone from *Ginkgo biloba* L., induces neuronal differentiation of cultured PC12 cells: potentiating the effect of nerve growth factor.

[9] Ginty D. D., Bonni A., Greenberg M. E. (1994). Nerve growth factor activates a Ras-dependent protein kinase that stimulates c-fos transcription via phosphorylation of CREB.

[10] Zhu Y., Duan X., Cheng X. (2016). Kai-Xin-San, a standardized traditional Chinese medicine formula, up-regulates the expressions of synaptic proteins on hippocampus of chronic mild stress induced depressive rats and primary cultured rat hippocampal neuron.

[11] Chen Z.-A., Wang J.-L., Liu R.-T. (2009). Liquiritin potentiate neurite outgrowth induced by nerve growth factor in PC12 cells.

[12] Schimmelpfeng J., Weibezahn K.-F., Dertinger H. (2004). Quantification of NGF-dependent neuronal differentiation of PC-12 cells by means of neurofilament-L mRNA expression and neuronal outgrowth.

[13] Lam K. Y. C., Chen J., Lam C. T. W. (2016). Asarone from Acori tatarinowii Rhizoma potentiates the nerve growth factor-induced neuronal differentiation in cultured PC12 cells: a signaling mediated by protein kinase A.

[14] Ishii T., Itoh K., Yamamoto M. (2002). Roles of Nrf2 in activation of antioxidant enzyme genes via antioxidant responsive elements.

[15] Lee J. M., Calkins M. J., Chan K., Kan Y. W., Johnson J. A. (2003). Identification of the NF-E2-related factor-2-dependent genes conferring protection against oxidative stress in primary cortical astrocytes using oligonucleotide microarray analysis.

[16] Lee J.-M., Johnson J. A. (2004). An important role of Nrf2-ARE pathway in the cellular defense mechanism.

[17] Hu Y., Liao H.-B., Dai-Hong G., Liu P., Wang Y.-Y., Rahman K. (2010). Antidepressant-like effects of 3,6′-disinapoyl sucrose on hippocampal neuronal plasticity and neurotrophic signal pathway in chronically mild stressed rats.

[18] Naidu M., Kuan C.-Y. K., Lo W.-L. (2007). Analysis of the action of euxanthone, a plant-derived compound that stimulates neurite outgrowth.

[19] Lee J.-H., Lee Y. J., Shin J.-K. (2009). Effects of triterpenoids from *Poria cocos* Wolf on the serotonin type 3A receptor-mediated ion current in *Xenopus oocytes*.

[20] Kim H. D., Jeong K. H., Jung U. J., Kim S. R. (2016). Naringin treatment induces neuroprotective effects in a mouse model of Parkinson's disease in vivo, but not enough to restore the lesioned dopaminergic system.

[21] Oztanir M. N., Ciftci O., Cetin A., Aladag M. A. (2014). Hesperidin attenuates oxidative and neuronal damage caused by global cerebral ischemia/reperfusion in a C57BL/J6 mouse model.

[22] Ho S.-L., Poon C.-Y., Lin C. (2015). Inhibition of *β*-amyloid aggregation by albiflorin, aloeemodin and neohesperidin and their neuroprotective effect on primary hippocampal cells against *β*-amyloid induced toxicity.

